# Influenza A (H1N1): the first hit for transfusion-related acute lung injury?

**DOI:** 10.1186/cc13304

**Published:** 2014-03-17

**Authors:** F Piza, F Carvalho, H Li, T Crochemore, R Rodrigues

**Affiliations:** 1 Hospital Israelita Albert Einstein, São Paulo, Brazil; 2 Hospital das Clinicas da Faculdade de Medicina da USP, São Paulo, Brazil

## Introduction

Transfusion-related acute lung injury (TRALI) is the leading cause of transfusion-related fatalities [1]. A two-hit hypothesis has been proposed for TRALI. The first hit is underlying patient factors, resulting in adherence of primed neutrophils to the pulmonary endothelium, such as severe pneumonia due to influenza A H1N1. The second hit is caused by mediators in the blood transfusion that activate the endothelial cells and pulmonary neutrophils, resulting in capillary leakage and subsequent pulmonary edema [2]. TRALI is a clinical diagnosis with the following criteria: acute onset within 6 hours of blood transfusion, PaO_2_/FIO_2 _ratio <300 mmHg, or worsening of the P:F ratio, bilateral infiltrative changes on chest radiograph, no sign of hydrostatic pulmonary edema (pulmonary arterial occlusion pressure ≤18 mmHg or central venous pressure ≤15 mmHg) and no other risk factor for acute lung injury [2].

## Methods

We describe a fatal TRALI in a patient with influenza A (H1N1), suggesting a relationship between a first-hit lung injury followed by the second lung impairment after blood transfusions.

## Results

We report a 57-year-old female, without a previous medical record. She had an acute onset of fever, cough, muscle pain and progressive dyspnea leading to acute respiratory distress syndrome. The test for influenza A H1N1 was positive. She was recovering, but on day 12 of admission, after 1 hour of platelet transfusion, she started with intensive tachycardia, dyspnea and hypoxemia. Her mechanical ventilation parameters increased dramatically. She was in plan for extubation with FiO_2 _of 30% and positive end-expiratory pressure of 8, which became 100% and 14 respectively. The P:F ratio dropped to 62. Her leukocytes were 10.6 × 10^9^/l a few hours earlier and went down to 1.5 × 10^9^/l after the onset. Previous lactate was normal, but jumped to 42 mg/dl. She was free of vasopressors and after the offending transfusion went through refractory shock and died approximately 24 hours after the blood transfusion.

## Conclusion

For our knowledge this is the first case reported of TRALI in an influenza A (H1N1) patient. Although blood transfusion can be life saving, it also can be a life-threatening intervention. Prevention is still the best hit.
